# Oppurtunistic completion of the 9 diabetic care processes during inpatient admission to a mental health hospital: an audit of local practice

**DOI:** 10.1192/bjo.2021.221

**Published:** 2021-06-18

**Authors:** Chad Brooker-Thompson, Yasmin Sultana, Adeela Ashraf

**Affiliations:** Goodmayes Hospital, North East London NHS Foundation Trust

## Abstract

**Aims:**

Diabetes is more prevalent in people with mental illness than in the general population. Those with both mental illness and diabetes are more likely to have poor glycaemic control. Clients with mental illness and diabetes are less likely to receive the 9 NICE recommended annual diabetic care processes than the general population. In 2017, the Joint British Diabetes Societies for Inpatient Care (JBDS-IP) and the Royal College of Psychiatrists released guidance recommending that inpatient psychiatric admissions should be used as an opportunity to complete diabetic care processes, and a named staff member should be responsible for this.

We aimed to review local compliance with this JBDS-IP guidance, increase knowledge and improve local care for clients living with both mental illness and diabetes.

**Method:**

We reviewed the notes of all current inpatients to general, forensic or learning disability wards at our centre and identified all patients with a known diagnosis of Diabetes. We identified which of the 9 care processes had been completed (or had the most recent result documented, or had a plan made for completion) during this admission. We identified if a named staff member was responsible for completing processes on each ward, and whether the care processes were documented in the patients’ notes.

**Result:**

We identified 18 current diabetic inpatients at our centre (14% of inpatients). We found that none of these patients had a diabetic care processes review documented and none of these patients had had a foot check and urinary albumin performed during admission, or had the last community result identified and documented. We found that less than 15% of patients had a documented plan concerning the completion of retinal screening. One ward had a named staff member responsible for reviewing their diabetic patients’ screening. However, 6/9 care processes had been completed in the significant majority of patients (>75%).

**Conclusion:**

Our centre is not compliant with the guideline audited. We have implemented a plan to increase awareness of care processes through posters, teaching (at junior and consultant level), creating documentation templates and ensuring wards nominate a staff member to review care processes. We have organised a re-audit. Organising foot examination, renal function testing and retinal screening during admission for clients who may have complicated social situations and may not be aware of (or be non adherent with) the long term management of their diabetes has the potential to significantly reduce morbidity in this client subgroup.

